# Lack of CFTR in Skeletal Muscle Predisposes to Muscle Wasting and Diaphragm Muscle Pump Failure in Cystic Fibrosis Mice

**DOI:** 10.1371/journal.pgen.1000586

**Published:** 2009-07-31

**Authors:** Maziar Divangahi, Haouaria Balghi, Gawiyou Danialou, Alain S. Comtois, Alexandre Demoule, Sheila Ernest, Christina Haston, Renaud Robert, John W. Hanrahan, Danuta Radzioch, Basil J. Petrof

**Affiliations:** 1 Meakins-Christie Laboratories and Respiratory Division, McGill University Health Centre and Research Institute, Montreal, Quebec, Canada; 2 Université Paris 6 Pierre et Marie Curie, UPRES EA2397, Paris, France; 3 Department of Physiology, McGill University, Montreal, Quebec, Canada; 4 Centre for the Study of Host Resistance, Departments of Experimental Medicine and Human Genetics, McGill University Health Centre Research Institute, Montreal, Quebec, Canada; The Jackson Laboratory, United States of America

## Abstract

Cystic fibrosis (CF) patients often have reduced mass and strength of skeletal muscles, including the diaphragm, the primary muscle of respiration. Here we show that lack of the CF transmembrane conductance regulator (CFTR) plays an intrinsic role in skeletal muscle atrophy and dysfunction. In normal murine and human skeletal muscle, CFTR is expressed and co-localized with sarcoplasmic reticulum-associated proteins. CFTR–deficient myotubes exhibit augmented levels of intracellular calcium after KCl-induced depolarization, and exposure to an inflammatory milieu induces excessive NF-kB translocation and cytokine/chemokine gene upregulation. To determine the effects of an inflammatory environment in vivo, sustained pulmonary infection with *Pseudomonas aeruginosa* was produced, and under these conditions diaphragmatic force-generating capacity is selectively reduced in *Cftr* ^−/−^ mice. This is associated with exaggerated pro-inflammatory cytokine expression as well as upregulation of the E3 ubiquitin ligases (MuRF1 and atrogin-1) involved in muscle atrophy. We conclude that an intrinsic alteration of function is linked to the absence of CFTR from skeletal muscle, leading to dysregulated calcium homeostasis, augmented inflammatory/atrophic gene expression signatures, and increased diaphragmatic weakness during pulmonary infection. These findings reveal a previously unrecognized role for CFTR in skeletal muscle function that may have major implications for the pathogenesis of cachexia and respiratory muscle pump failure in CF patients.

## Author Summary

Cystic fibrosis is an autosomal recessive disorder caused by mutations of the CF transmembrane conductance regulator (CFTR), which acts as a chloride channel and also participates in the regulation of other ions and proteins. In most CF patients, the clinical course is dominated by lung disease and recurrent pulmonary bacterial infections. Many CF patients also have significant skeletal muscle wasting and weakness, and this can affect the most essential breathing muscle, the diaphragm. Although muscle wasting in CF has generally been attributed to factors such as reduced physical activity and poor nutrition, our study reveals an intrinsic defect of skeletal muscle function caused by the lack of CFTR. Hence, we show that CFTR is normally found in skeletal muscle fibers of humans and mice. In CF muscle cells lacking CFTR, abnormal elevations of calcium and inflammatory gene expression are found. In addition, during pulmonary infections, diaphragm muscles lacking CFTR show greater weakness and induction of genes which cause muscle atrophy. These findings extend our understanding of the factors leading to exercise limitation and disability in CF and also have implications for the pathogenesis of respiratory muscle failure in CF patients during lung infections.

## Introduction

Cystic fibrosis (CF) is caused by defects in the gene encoding the cystic fibrosis transmembrane conductance regulator (CFTR) [1]. Despite improvements in therapy, a progressive loss of lung function, associated with recurrent pulmonary infections and inflammation, still prevails. Over time, the diaphragm and other respiratory muscles become unable to sustain the increased breathing requirement, thereby leading to respiratory failure. This ultimately fatal situation is favored by the presence of generalized skeletal muscle atrophy (cachexia) and weakness in many CF patients, which has been attributed to malnutrition, mechanical disadvantage of the respiratory muscles, and other factors [2],[3].

CFTR is a member of the superfamily of ATP-binding cassette (ABC) transporter ATPases, which functions as a cAMP-regulated chloride (Cl^−^) channel [1],[4]. The actions of CFTR extend beyond Cl^−^channel function, with several other roles described in the secretion or absorption of different ions as well as in the transport or regulation of other macromolecules [5],[6]. It is also reported that cells with defective CFTR have a hyperinflammatory phenotype, characterized by an excessive production of cytokines triggered by various stimuli [7]–[11]. The exaggerated inflammatory response in CFTR-defective cells has been linked to enhanced activation of the transcription factor NF-kB [7], [9]–[12], a central regulator of inflammation.

Intriguingly, CFTR transcripts have been detected in rat skeletal muscle, although no attempts were made to elucidate a functional role [13]. However, exercise performance can be impaired in CF patients at early stages of the disease despite essentially normal pulmonary function and nutritional status [14],[15]. In addition, exercise limitation in CF patients appears to differ according to the type of CFTR mutation, independent of pulmonary function [16]. These and other observations have led to speculation that a specific CFTR-related functional defect could exist in the skeletal muscles of CF patients [17].

In the present study, we sought to determine whether a specific link exists between CFTR and skeletal muscle function, with a particular emphasis on the diaphragm due to its importance for survival. Our primary objectives were two-fold: 1) to confirm the presence, localization, and functionality of CFTR in normal skeletal muscle; and 2) to determine whether CFTR deficiency predisposes to diaphragmatic muscle inflammation, atrophy, or weakness. Here we report that CFTR protein is normally found at the level of the sarcoplasmic reticulum (SR) in skeletal muscle, where it appears to modulate intracellular calcium fluxes. We also show that in the setting of lung infection, the diaphragms of *Cftr* ^−/−^ mice have higher levels of pro-inflammatory gene expression, an increased induction of cachexia-inducing components of the ubiquitin-proteasome pathway, and a more severe impairment of force-generating capacity than wild-type controls. Taken together, these findings point to an intrinsic alteration of skeletal muscle function in CFTR deficiency, which is manifested by greater cytokine expression, atrophy, and force loss upon exposure to an inflammatory milieu. In view of the increased pulmonary and systemic inflammation found in CF, these phenomena could play a major role in promoting chronic cachexia, as well as the development of respiratory muscle failure during acute pulmonary infections in CF patients.

## Results

### Expression of CFTR in murine and human skeletal muscle

**Figure 1:**
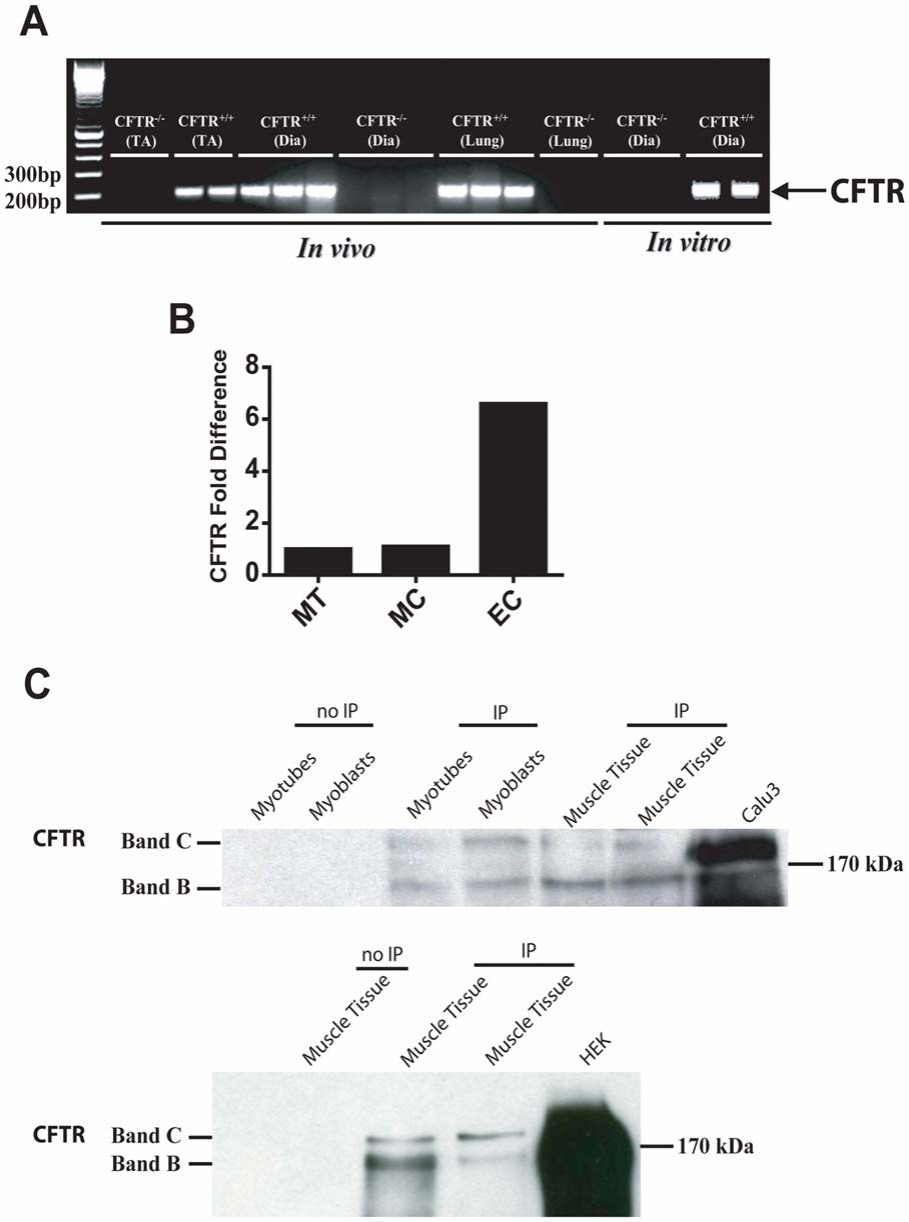
Expression of CFTR in normal skeletal muscle. (A) CFTR transcripts in lung, tibialis anterior (TA), and diaphragm (Dia) muscle tissue (in vivo) as well as in cultured myotubes from wild-type (*Cftr^+/+^*) mouse diaphragms (in vitro). (B) Relative quantification of human CFTR mRNA in skeletal muscle tissue (MT), cultured E6/E7 skeletal muscle cells (MC), and cultured epithelial cells (EC) from human nasal mucosa. (C) Western blotting for human CFTR after immunoprecipitation (IP) of CFTR protein in human skeletal muscle tissue and cultured human muscle cells (myotubes and myoblasts), as well as CFTR-overexpressing positive control cells (Calu3 or HEK). Note the presence of both mature (Band C) and immature (Band B) forms of CFTR. Top Panel: lysate amounts for IP were 1000 (lanes 3–5), 2000 (lane 6), and 200 µg (lane 7). Bottom Panel: lysate amounts for IP were 3000, 1500, and 1000 µg for lanes 2–4, respectively.

We first demonstrated CFTR mRNA expression in diaphragmatic and limb muscle tissues obtained from wild-type (*Cftr+/+*) mice, and this was further confirmed in primary diaphragmatic myotube cultures (see Figure 1A). As CFTR expression is classically associated with epithelial cells, we then compared CFTR expression in epithelial and skeletal muscle samples. Real-time PCR revealed that CFTR mRNA levels in both human skeletal muscle tissue and cultured muscle cells were approximately 7-fold lower than in human nasal epithelial cell cultures (Figure 1B). Furthermore, immunoprecipitation followed by western blot analysis confirmed the presence of CFTR protein in human skeletal muscle tissue and in cultured muscle cells (Figure 1C).

**Figure 2:**
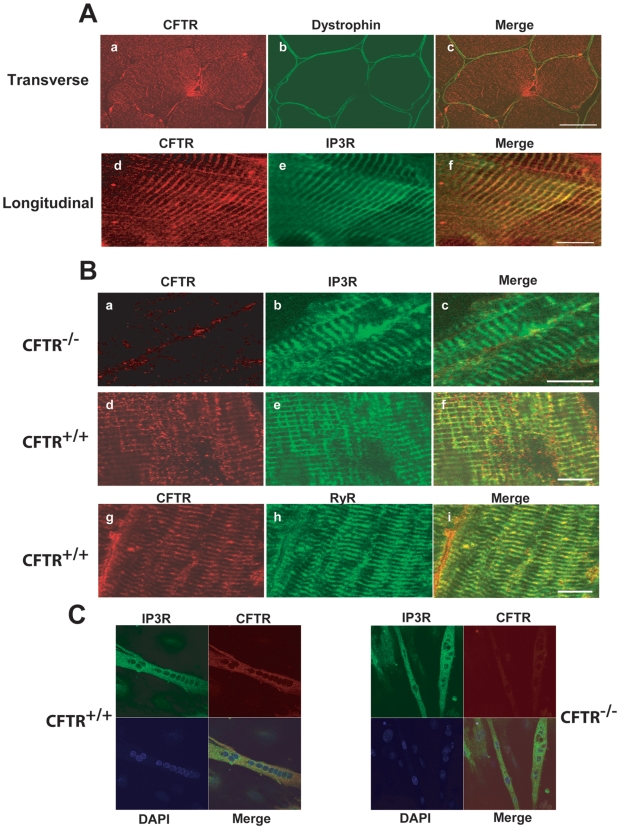
Confocal localization of CFTR in skeletal muscle. (A) Double labeling for CFTR and either membrane- (dystrophin) or SR- (IP3R) associated proteins in transverse and longitudinal sections of human skeletal muscle. (B) Double labeling of CFTR and SR (IP3R or RyR) proteins in longitudinal sections of *Cftr+/+* and *Cftr−/−* mouse skeletal muscle (scale bar = 10 µm). (C) Triple labeling of IP3R, CFTR, and myonuclei (DAPI) in cultured mouse myotubes.

Confocal immunofluorescence labeling of CFTR together with either dystrophin or inositol triphosphate receptors (IP3Rs) – markers of the sarcolemma and sarcoplasmic reticulum (SR), respectively – were used to localize the CFTR protein. On transverse cross-sections of human skeletal muscle, only occasional surface membrane staining for CFTR could be demonstrated (Figure 2A, a–c). However, longitudinal sections showed a consistent pattern of stereotypical periodicity that co-localized to a substantial degree with IP3R immunostaining (Figure 2A, d–f), suggesting that CFTR is associated with the SR. These findings were reproduced in murine skeletal muscle (Figure 2B, d–f), and CFTR was further co-localized with another SR protein, the ryanodine receptor (RyR) (Figure 2B, g–i). As expected, no CFTR staining was observed in the muscles of *Cftr−/−* mice (Figure 2B, a–c). Furthermore, in myotubes derived from *Cftr+/+* mice, co-localization of CFTR with IP3Rs was also demonstrated (Figure 2C).

### *Cftr* ^−/−^ muscle cells exhibit abnormal calcium regulation

**Figure 3:**
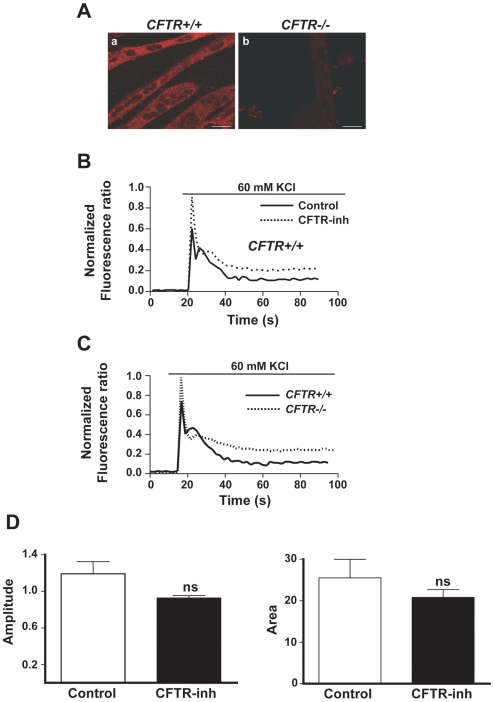
Abnormal calcium responses in CFTR–deficient muscle cells. (A) Negative immunostaining for CFTR in *Cftr* ^−/−^ myotubes. (B) Representative Ca^2+^ responses in *Cftr^+/+^* myotubes, either without (control) or with (CFTR-inh) pre-treatment by CFTR-inh172. (C) Representative Ca^2+^ responses in *Cftr^+/+^* versus *Cftr* ^−/−^ myotubes. (D) Lack of effect of CFTR-inh172 on the area under the curve or peak amplitude of Ca^2+^ responses in *Cftr* ^−/−^ myotubes.

**Figure 4:**
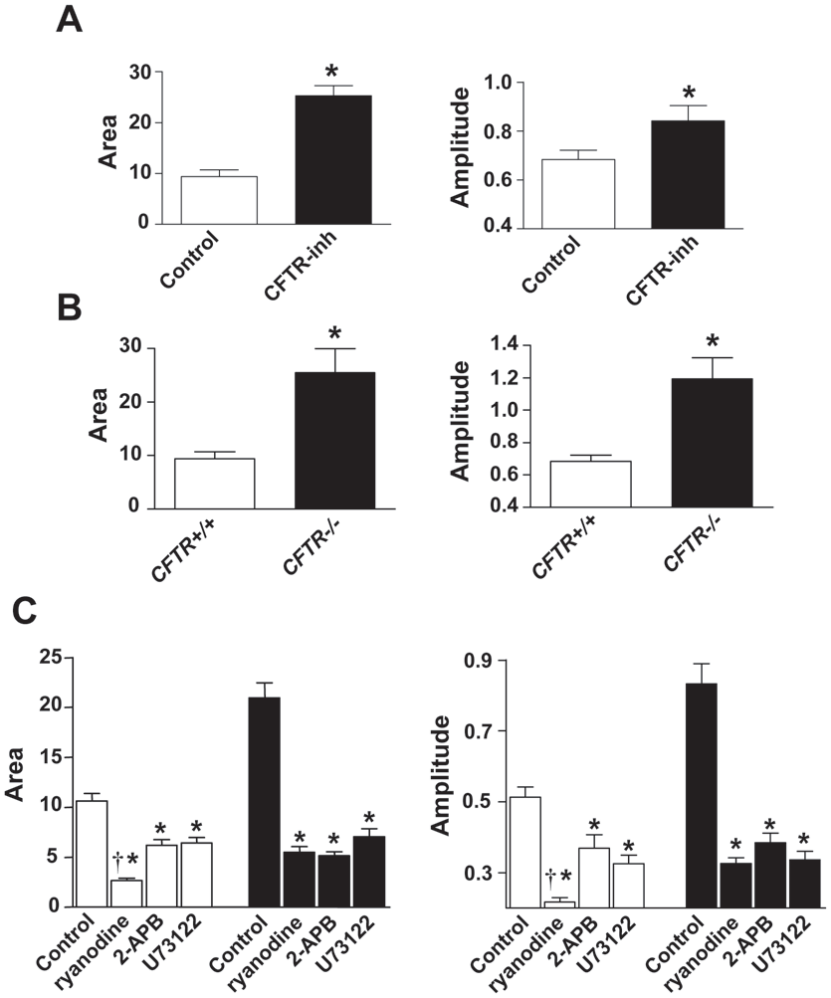
Quantification and inhibition of SR–mediated calcium release. (A) Group mean data for intracellular Ca^2+^ responses in *Cftr^+/+^* myotubes, either without (control) or with (CFTRinh) pre-treatment by CFTR-inh172. (B) Group mean data for intracellular Ca^2+^ responses in *Cftr^+/+^* versus *Cftr* ^−/−^ myotubes. (C) Effects of inhibiting RyR or IP3R function in *Cftr^+/+^* (open bars) and *Cftr* ^−/−^ (filled bars) myotubes. For all experiments, N = minimum of 30 myotubes per group. * p<0.05 versus control, † p<0.05 for ryanodine versus 2-APB and U73122.

The SR in skeletal muscle is the major source of Ca^2+^ released via both RyRs and IP3Rs following muscle cell depolarization [18]–[20]. To determine whether CFTR has an influence upon this process, cultured *Cftr^+/+^* and *Cftr* ^−/−^ myotubes were loaded with the Ca^2+^ probe Fura-2 and exposed to high potassium (60 mM KCl) to induce depolarization in vitro. Immunostaining confirmed the absence of intracellular staining for CFTR in *Cftr−/−* myotubes (Figure 3A). In *Cftr^+/+^* myotubes, the Ca^2+^ response consisted of a rapid rise to a peak amplitude followed by a progressive decline to a plateau level, as shown in Figure 3B (solid line). When *Cftr^+/+^* myotubes were pre-treated with a small molecule inhibitor of CFTR channel activity (CFTR-inh172), there was a greater initial peak as well as a higher plateau value (Figure 3B, dashed line). Quantitative analysis confirmed that the area under the Ca^2+^ versus time curve and the peak amplitude of Ca^2+^ responses were significantly greater in *Cftr^+/+^* myotubes treated with the CFTR inhibitor (see Figure 4A). An identical approach was used to compare Ca^2+^ responses of *Cftr^+/+^* and *Cftr* ^−/−^ myotubes. The representative tracings in Figure 3C and group mean quantitative analyses in Figure 4B show that *Cftr* ^−/−^ myotubes responded in a very similar fashion to *Cftr^+/+^* myotubes treated with CFTR-inh172. No differences were detected between *Cftr^+/+^* and *Cftr* ^−/−^ myotubes with respect to their total SR Ca^2+^ content (evaluated by blocking Ca^2+^ re-uptake into the SR with thapsigargin), nor were the results influenced by omission of Ca^2+^ from the extracellular medium (data not shown). Furthermore, in *Cftr* ^−/−^ muscle cells, pre-treatment with CFTR-inh172 had no effect on Ca^2+^ responses (Figure 3D).

To better understand how CFTR could interact with RyRs and/or IP3Rs, ryanodine was used to interfere with RyR function, whereas for IP3 pathway interference two different compounds, 2-APB and U73122, were used as inhibitors of IP3Rs and phospholipase C, respectively. As shown in Figure 4C, in both *Cftr^+/+^* and *Cftr* ^−/−^ myotubes all of these treatments greatly reduced the area under curve as well as the peak amplitude of the Ca^2+^ response to depolarization. However, in *Cftr^+/+^* myotubes the inhibition of RyR function had more pronounced effects than interference with the IP3 pathway. On the other hand, RyR and IP3R blockade had equivalent impacts upon intracellular Ca^2+^ in *Cftr* ^−/−^ myotubes, providing further evidence that Ca^2+^ mobilization is dysregulated in CFTR-deficient muscle cells.

### *Cftr* ^−/−^ muscle cells show a hyperinflammatory phenotype in vitro

**Figure 5:**
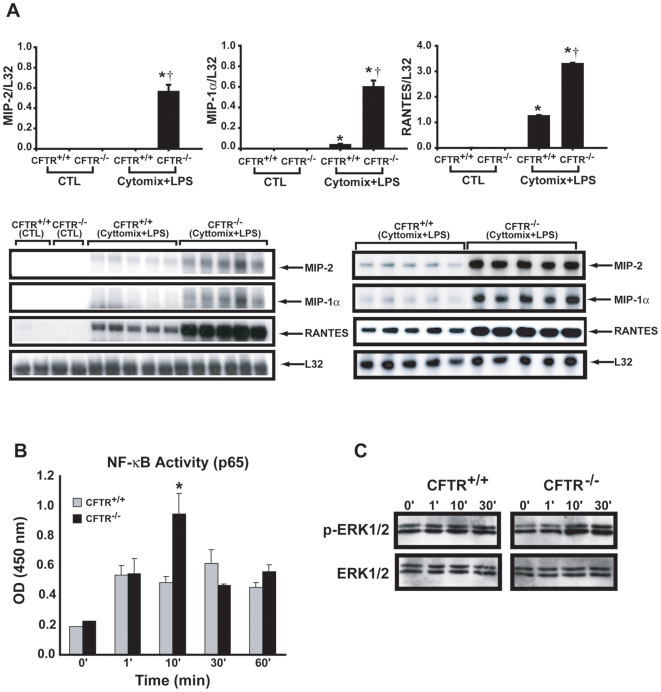
Hyperinflammatory phenotype of *Cftr−/−* skeletal muscle cells in vitro. (A) Upper panels: Cytokine mRNA expression levels in *Cftr^+/+^* versus *Cftr* ^−/−^ diaphragmatic myotubes under control conditions (CTL) and after 4 hours of stimulation (TNF-α 1 ng/ml, IL-1α 5 U/ml, IFN-γ 20 U/m, LPS 1 ng/ml). N = 5 per group; * p<0.05 for control versus stimulated groups, † p<0.05 for *Cftr^+/+^* versus *Cftr* ^−/−^. Lower panels: Representative RNase protection assays from diaphragm (left panel) and limb muscle (right panel). (B) NFκB p65 levels in nuclear extracts (normalized for protein) obtained from diaphragmatic myotubes in *Cftr^+/+^* versus *Cftr* ^−/−^ groups stimulated for the indicated time periods (0–60 min). N = 4–5 per group; * p<0.05 for *Cftr^+/+^* versus *Cftr* ^−/−^. (C) Western analysis of phosphorylated (pERK1/2) and total ERK1/2 MAPK in cytoplasmic lysates from *Cftr^+/+^* and *Cftr* ^−/−^ diaphragmatic myotubes following stimulation with cytokines+LPS. Results are representative of 2 experiments.

CFTR defects have been associated with a hyperinflammatory phenotype, with IL-8 in particular having been implicated [7], [9]–[11]. Therefore, we compared MIP-2 (murine analogue of human IL-8) expression in *Cftr^+/+^* and *Cftr* ^−/−^ muscle cells. There were no detectable differences between wild-type and *Cftr* ^−/−^ myotubes under basal conditions. However, after stimulation with pro-inflammatory cytokines (TNF-α, IL-1α, IFN-γ) and *Pseudomonas* LPS, MIP-2 showed greater upregulation in *Cftr* ^−/−^ diaphragmatic myotubes. This increased responsiveness also involved other pro-inflammatory chemokines such as MIP-1α and RANTES, and was similarly observed in limb muscle-derived myotubes (Figure 5A). Because many pro-inflammatory genes are regulated by NF-kB, and since the latter has been implicated in the hyperinflammatory phenotype of CF epithelial cells, we examined NF-kB (p65) binding to its DNA consensus site in nuclear extracts obtained from *Cftr^+/+^* and *Cftr* ^−/−^ diaphragmatic myotubes. As shown in Figure 5B, greater binding of the NF-kB p65 subunit was observed in *Cftr* ^−/−^ diaphragm muscle cells at 10 min after initiating stimulation. In addition, inhibition of the NF-kB pathway by pre-treatment with the IkB kinase (IKK)-2 inhibitor SC-514 had similar effects in downregulating MIP-1α mRNA expression in *Cftr* ^−/−^ and *Cftr^+/+^* myotubes (3–4 fold reductions for both; p<0.05, n = 6 per group), but it was only effective in doing so for MIP-2 (2.4 fold; p<0.05) and RANTES (5.8 fold; p<0.05) in *Cftr* ^−/−^ myotubes, suggesting a greater reliance upon this pathway in the latter. Finally, we observed that ERK1/2 MAPK, another signal transduction molecule implicated in NF-kB activation and cytokine gene upregulation [20], also showed greater phosphorylation following cytomix/LPS stimulation in the *Cftr* ^−/−^ group (Figure 5C).

### Exaggerated pro-inflammatory and atrophy gene responses in vivo

**Figure 6:**
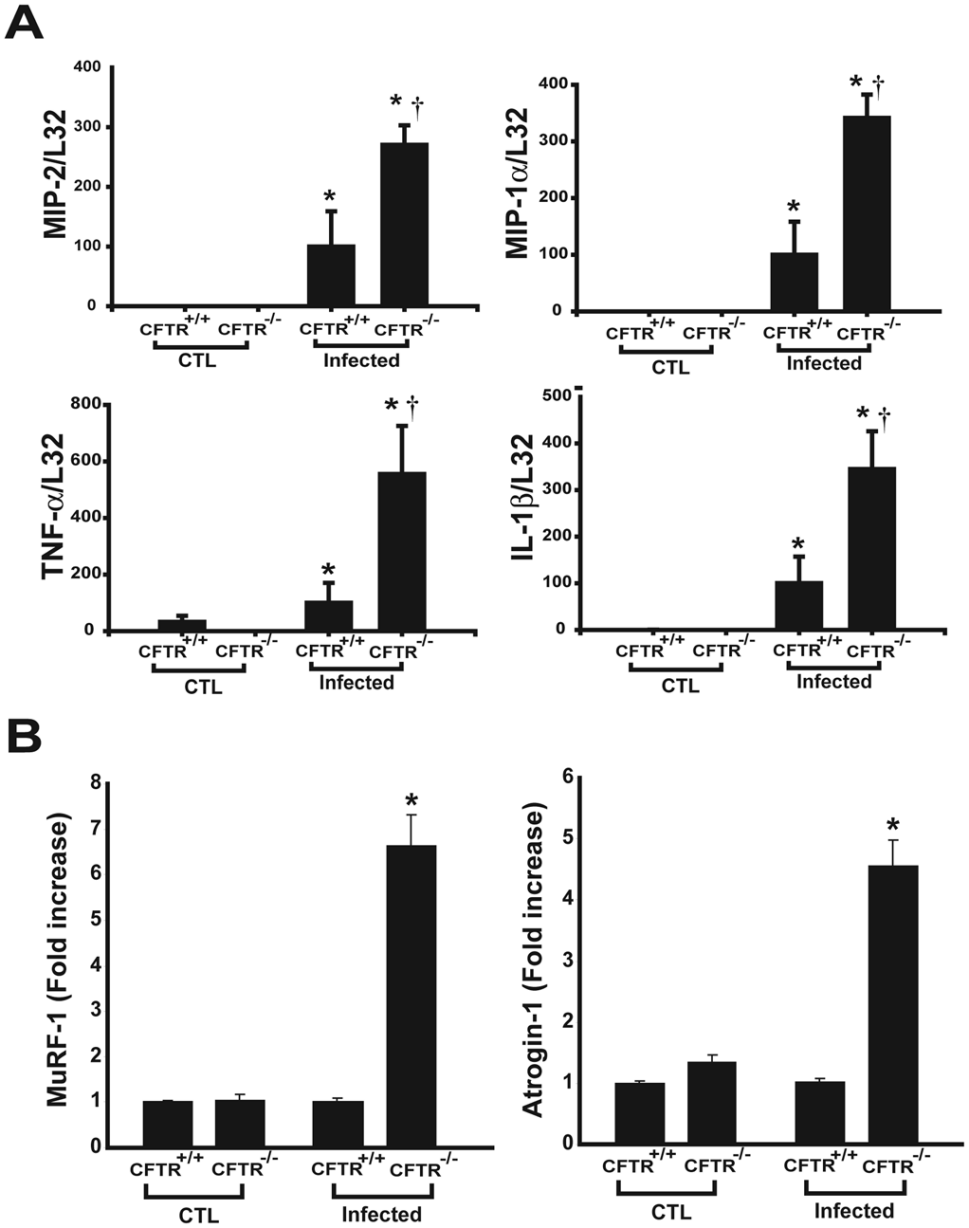
Hyperinflammatory phenotype of *Cftr^−/−^* skeletal muscle in vivo. (A) Cytokine mRNA expression levels in *Cftr^+/+^* versus *Cftr* ^−/−^ diaphragms for uninfected (CTL) and *Pseudomonas*-infected (day 2) mice. N = 4–5 mice per group; * p<0.05 for control versus infected mice, † p<0.05 for *Cftr^+/+^* versus *Cftr* ^−/−^. (B) Real-time PCR quantification of the E3 ubiquitin ligases MuRF-1 and atrogin-1 in diaphragms of control and infected mice. * p<0.05 for *Cftr^+/+^* versus *Cftr* ^−/−^.

To evaluate the potential relevance of the above findings to the *in vivo* context, *Cftr* ^−/−^ mice were infected with *P. aeruginosa*. We used the *Cftr* ^−/−^ strain (CFTR^tm1UNC^) backcrossed onto the C57Bl/6 background that has been shown to develop spontaneous lung disease [21]. As has been previously reported [22],[23], the pulmonary bacterial burden and number of inflammatory cells in bronchoalveolar lavage fluid were greater in infected *Cftr* ^−/−^ mice (data not shown). At 2 days after the initiation of pulmonary infection, there was an upregulation of pro-inflammatory gene expression within the diaphragms of both *Cftr* ^−/−^ and *Cftr* ^+/+^ mice. However, as was the case *in vitro*, the diaphragmatic mRNA levels of chemokines (MIP-2, MIP-1α, RANTES) and other cytokines (TNF-α, IL-1β) were significantly higher in infected *Cftr* ^−/−^ mice (Figure 6A). Therefore, the greater vulnerability of *Cftr* ^−/−^ diaphragms to developing a hyperinflammatory phenotype *in vitro* was also observed *in vivo*.

Because pro-inflammatory cytokines have been linked to muscle wasting via the ubiquitin-proteasome system, we proceeded to measure the diaphragmatic expression levels of two key regulators of this proteolytic pathway in skeletal muscle, MuRF-1 and atrogin-1 [24]. Diaphragmatic expression levels of both MuRF-1 and atrogin-1 were increased in *Cftr* ^−/−^ mice, but not in their wild-type littermates, after pulmonary infection with *P. aeruginosa* (Figure 6B). Measurements of limb muscle (soleus) weights also indicated a significant difference between infected *Cftr* ^−/−^ and *Cftr* ^+/+^ mice (6.48±0.17 mg versus 8.20±0.24 mg; p<0.0001, n = 7 per group). Therefore, these findings are consistent with a greater susceptibility of infected *Cftr* ^−/−^ mouse skeletal muscles not only to cytokine upregulation, but also to ubiquitin-mediated proteolysis and atrophy.

### Altered contractile function and excessive force loss during *P. aeruginosa* lung infection in diaphragms of *Cftr* ^−/−^ mice

**Figure 7:**
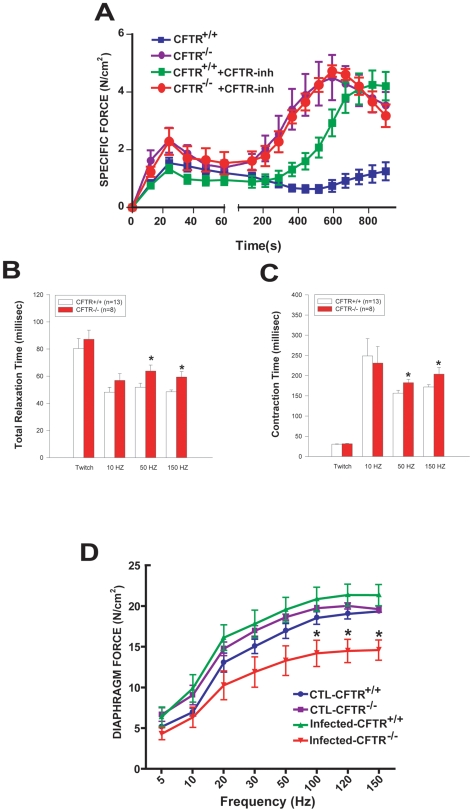
Effects of CFTR status on diaphragmatic contractile function. (A) Response of intact diaphragmatic muscle fibers to KCl-induced depolarization in *Cftr^+/+^* versus *Cftr* ^−/−^ groups, under basal conditions (N = 6–7 mice per group) or with CFTR- inh172 (N = 4–5 mice per group) added to the perfusate (100 µM). (B,C): Effects of CFTR deficiency on relaxation and contraction times of the diaphragm during electrical stimulation (white bars: *Cftr^+/+^*; red bars: *Cftr* ^−/−^). (D) Differential effects of *Pseudomonas* lung infection on diaphragmatic force capacity in *Cftr^+/+^* and *Cftr* ^−/−^ mice (N = 5 mice per group). * p<0.05 for *Cftr^+/+^* versus *Cftr* ^−/−^.

We initially examined whether the different KCl-evoked responses of cultured *Cftr^+/+^* and *Cftr* ^−/−^ myotubes could be ascertained in intact perfused diaphragmatic muscle fibers. Figure 7A shows major differences in the force response profiles of *Cftr^+/+^* (monophasic pattern) and *Cftr* ^−/−^ (biphasic pattern) fibers during KCl-induced contraction. Moreover, *Cftr^+/+^* diaphragms incubated with CFTR-inh172 exhibited a biphasic pattern similar to the *Cftr* ^−/−^ group, while CFTR-inh172 had no effect upon *Cftr* ^−/−^ diaphragms. In addition, when diaphragmatic fibers from *Cftr* ^−/−^ were electrically stimulated, a significant prolongation of relaxation and contraction were observed during tetanic (but not single twitch) stimulation as compared to *Cftr^+/+^* diaphragms (Figure 7B and 7C). Taken together, these results corroborate the abnormal responses to depolarization in *Cftr* ^−/−^ myotubes and further indicate a functional alteration in intact skeletal muscle fibers lacking CFTR.

To determine whether the greater increases in pro-inflammatory and atrophy gene expression in *Cftr* ^−/−^ diaphragms are associated with exaggerated muscle weakness during pulmonary infection, force measurements were performed on electrically stimulated fibers. As shown in Figure 7D, lung infection had adverse effects upon diaphragmatic force production in the *Cftr* ^−/−^ group only. Hence at the *P. aeruginosa* inoculation dose of 1×10^5^ cfu employed, force-generating capacity of *Cftr^+/+^* diaphragms remained normal, whereas *Cftr* ^−/−^ diaphragms generated maximal force values which were approximately one-third lower than in identically infected *Cftr^+/+^* mice. Taken together, these results indicate that the pro-inflammatory and atrophy gene expression profiles found in the diaphragms of infected *Cftr* ^−/−^ mice are associated with a significant impairment of contractile function, which is manifested by profound weakness of this critically important respiratory muscle.

## Discussion

The results of our study demonstrate a previously unrecognized role for CFTR in skeletal muscle physiology, and supply new insights into a potential mechanism favoring the development of respiratory muscle failure in CF. The main findings of our investigation can be summarized as follows: 1) CFTR is normally expressed at the SR of skeletal muscle fibers and is clearly functional, as indicated by abnormal intracellular Ca^2+^ and contractile responses to depolarization in muscles with CFTR deficiency; and 2) diaphragm muscles lacking CFTR exhibit an exaggerated upregulation of cytokines and cachexia-inducing factors during pulmonary infection, which is associated with a major deterioration of diaphragmatic function.

### CFTR expression and function in normal skeletal muscle

CFTR expression has been reported in both cardiac [25]–[27] and smooth muscle [28]. To our knowledge, there is only one prior report of CFTR expression in skeletal muscle, in which a single mRNA species of 7.5 kb was demonstrated in rat skeletal muscle, as compared to the 6.3 kb transcript found in the lung and intestines [13]. Whereas the most common mutation (F508 delta) in CF patients causes CFTR to be retained in the endoplasmic reticulum of epithelial cells, our results suggest that this may be the major location of CFTR in skeletal muscle. Although we do not exclude a co-existent surface membrane localization, in our study CFTR was mostly co-localized with RyRs and IP3Rs in their characteristic cross-striated staining pattern [18]. This is reminiscent of ClC-4, which co-localizes at the surface membrane with CFTR in intestinal epithelial cells [29] but was recently shown to be highly expressed in the SR of skeletal muscle [30]. While the specific protein motifs responsible for differential cellular localization of the epithelial and skeletal muscle isoforms of CFTR are unknown, the CFTR gene is known to exhibit complex patterns of tissue-specific expression, with different transcription start sites and alternative splicing [27],[31].

In skeletal muscle, the ClC-1 Cl^−^ channel at the surface membrane plays the predominant role in maintaining the normal resting electrical potential of the cell [32]. The normal physiological role of SR Cl^−^ channels is more ill-defined. Several Cl^−^ channels with distinct Cl^−^ conductances, selectivities, and voltage-dependencies have been characterized in SR vesicles [33],[34]. However, in most cases the specific molecular identities of these channels have not been determined. Both Ca^2+^-induced and voltage-activated Ca^2+^ release are enhanced by Cl^−^ exposure, apparently via increased activation of RyRs [33],[35]. However, Cl^−^ was also shown to inhibit caffeine-induced Ca^2+^ release [36], indicating that the regulatory effects of Cl^−^ channels upon SR function are complex. One proposed function for SR Cl^−^ channels is to permit Cl^−^ to act as a counter ion during high rates of Ca^2+^ release and uptake from the SR [33],[34], in order to restore the SR membrane potential, and it is possible that CFTR plays a role in this process. CFTR could also modulate SR function in a manner which is unrelated to Cl^−^ channel activity per se. For example, CFTR mediates glutathione flux in epithelial cells [5] and thus plays a role in controlling the redox state, which is an important modifier of SR Ca^2+^ release [37].

### Dysregulated calcium homeostasis in CFTR–deficient muscle

In the present study, we found that defective CFTR function is associated with increased Ca^2+^ mobilization in myotubes stimulated by potassium-induced depolarization. These findings are consistent with previous observations in *Cftr* ^−/−^ vascular smooth muscle, where greater KCl-induced vasoconstriction and impaired relaxation are found [28], as well as increased intracellular Ca^2+^ and enhanced vasoconstrictor responses during exposure to CFTR-inh172 [38]. In our study, Ca^2+^ responses to depolarization were similarly increased irrespective of whether CFTR function was inhibited by genetic or pharmacologic methods. Our data indicate that the SR is the primary source of this increased Ca^2+^ influx, since the latter was not affected by the removal of extracellular calcium. The fact that inhibiting the IP3-mediated pathway with either 2-APB or U73122 (but not the RyR) led to a decrease of Ca^2+^ values to levels obtained in *Cftr^+/+^* myotubes under the same conditions, suggests that the IP3R could be the most important source of dysregulated Ca^2+^ release from the SR in CFTR-deficient muscle cells, although this remains to be determined. In addition, experiments performed with thapsigargin, an inhibitor of the ER/SR Ca^2+^ re-uptake pump, did not point to an expansion of SR Ca^2+^ stores in *Cftr* ^−/−^ muscle cells as an explanation for the greater Ca^2+^ responses [39].

### Inflammatory/atrophic phenotype and force loss in CFTR–deficient muscle

Many studies have reported that CF cell lines as well as airway epithelial cells from CF patients exhibit exaggerated cytokine production [7],[8],[11]. Several possible explanations have been proposed, including greater Ca^2+^ release from the endoplasmic reticulum [40],[41]. Defective CFTR function has also been linked to hyperactivation of the NF-kB pathway as well as signaling molecules such as ERK1/2 MAPK [7], [9]–[12], which were demonstrated in the present study. Amongst the pro-inflammatory genes showing enhanced upregulation within CFTR-deficient skeletal muscle in this study, all are potential targets of NF-kB, and greater binding of the NF-kB p65 subunit was observed in *Cftr* ^−/−^ muscle cells.

There is also an accumulating literature implicating SR-mediated Ca^2+^ release, particularly from the IP3R, in the regulation of transcription factors controlling the expression of pro-inflammatory and other genes in normal skeletal muscle cells [42],[43]. In this regard, it was recently reported that depolarization-induced Ca^2+^ transients of even brief duration can activate NF-kB in myotubes [20]. Furthermore, this phenomenon has been linked to increased cytokine (IL-6) gene expression in skeletal muscle cells [44]. Therefore, although not directly examined in our study, it is plausible to speculate that the increased NF-kB activity and pro-inflammatory gene expression patterns observed in stimulated *Cftr* ^−/−^ muscle cells could be linked to greater Ca^2+^ release from the SR. Indeed, an analogous phenomenon of exaggerated Ca^2+^ release from the ER leading to NF-kB activation was recently reported in CF airway epithelial cells stimulated with IL-1β [41].

In the present study, we demonstrated two potential causes for muscle weakness in CFTR-deficient skeletal muscles. Firstly, we found that *Cftr* ^−/−^ mice are more prone to a loss of muscle mass when exposed to a hyperinflammatory milieu. This is likely linked at least in part to the observed upregulation of E3 ubiquitin ligases, MuRF-1 and atrogin-1, which are centrally involved in proteasomal degradation of muscle proteins atrophy [24]. Furthermore, NF-kB activation via IKK-2/β has been directly implicated in the induction of the ubiquitin ligases which cause muscle atrophy [24]. Secondly, even after correction for atrophy and reduced cross-sectional area of the muscle, diaphragmatic strength (specific force) was abnormally decreased in infected *Cftr* ^−/−^ mice. This suggests an alteration in the intrinsic contractile properties of the muscle above and beyond the loss of muscle mass. Recent work has suggested that caspase- and calpain-mediated breakdown of the contractile protein apparatus precedes protein degradation by the ubiquitin-proteasome system in sepsis, and may thus be responsible for the loss of specific force prior to atrophy [45],[46]. Indeed, the calpain system is activated by increases in intracellular Ca^2+^, and may therefore be particularly relevant to our findings [46]. Oxidative stress has also been strongly implicated in sepsis-induced reductions of diaphragmatic specific force [47] and it is interesting to note that *Cftr* ^−/−^ hearts are abnormally vulnerable to tissue damage caused by ischemia-reperfusion, a classical inducer of free radical-mediated injury [26].

### Clinical implications

Patients with CF most commonly die of type II (hypercapnic) respiratory failure, where the limiting factor is not oxygen transfer but rather the presence of respiratory muscle pump failure. This occurs when the load placed on the diaphragm exceeds its capacity to deal with the increased work of breathing. In earlier phases of the disease, the increased load may actually induce a beneficial training effect on the respiratory muscles [3]. However, diaphragmatic pressure generation normalized to its muscular mass can be reduced by as much as 40% even in clinically stable CF patients [3]. Therefore, any factor which further compromises the muscular strength of the diaphragm, such as an acute pulmonary infection with *P. aeruginosa*, will place patients at greater risk for the development of respiratory failure. In our study, CFTR-deficient skeletal muscles demonstrated a pathological phenotype primarily during exposure to an inflammatory milieu. This is likely to be clinically relevant, since studies suggest a quasi-constant exposure to abnormally high serum levels of inflammatory mediators in many CF patients [48], as well as a greater systemic inflammatory response to exercise [49].

**Figure 8:**
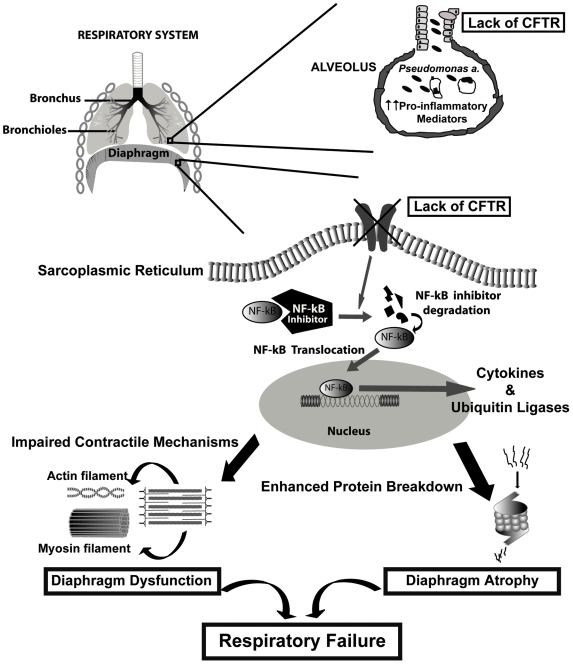
Schematic representation of respiratory muscle failure in CF. A greater inherent sensitivity of CF muscle cells to pro-inflammatory stimuli, when combined with the higher levels of pulmonary and systemic inflammation found in CF, leads to exaggerated NF-kB and cytokine activation within diaphragmatic muscle fibers. This in turn provokes diaphragmatic wasting and weakness in CF, thus contributing to the pathogenesis of respiratory failure.

Taken together, we believe our findings are consistent with a scenario (see Figure 8) in which two major factors act synergistically to favor the development of skeletal muscle dysfunction in CF: 1) a higher ambient level of pulmonary/systemic inflammation leading to greater exposure of the muscles to pro-inflammatory mediators; and 2) a greater inherent sensitivity of the muscles (related to their lack of CFTR) to the effects of pro-inflammatory mediator exposure. This can lead, in turn, to an exaggerated upregulation of atrophy pathways as well as an impairment of contractile mechanisms. On the other hand, when the level of systemic inflammation is normal or relatively low (e.g., in non-infected *Cftr* ^−/−^ mice and CF patients with early or mild disease), alterations in the function of CFTR-defective skeletal are more subtle, and may only be apparent with heavy usage such as during exercise [15].

In conclusion, the identification in this study of an intrinsic alteration of skeletal muscle function in CFTR deficiency extends our understanding of the factors leading to disability in CF patients. A major implication of our study is that the excessive levels of pulmonary and systemic inflammation found in patients with advanced CF can potentially initiate a positive feedback loop, in which the abnormal vulnerability of CFTR-deficient muscle to pro-inflammatory stimulation becomes an important component of the downward spiral leading to respiratory muscle failure. Our data also suggest that the chronic cachexia found in non-respiratory skeletal muscles of many CF patients could be related to similar mechanisms, and may thus be amenable to anti-inflammatory therapies.

## Materials and Methods

### Cell culture

Primary muscle cell cultures from the mouse diaphragm and limb muscle (tibialis anterior) were established using single living muscle fibers as previously described in detail [50]. Culture conditions for the E6/E7 human skeletal muscle cell line were as previously published [51]. Calu3 cells and HEK cells stably expressing CFTR were used as positive controls for western blot/immunoprecipitation studies.

### Immunofluorescence for confocal microscopy

Standard staining procedures were applied to myotubes grown on glass coverslips and frozen muscle tissue sections. The analysis was performed on 3 animals from the CFTR+/+ group, 2 animals from the CFTR−/− group, and 3 human muscle samples (3 sections each). Primary antibodies consisted of the following: 1∶100 dilution of anti-CFTR (clone 24–1, R&D Systems); 1∶50 dilution of anti-dystrophin (clone ab15277, Abcam); 1∶100 dilution of anti-IP3R (clone NR09, Calbiochem); 1∶50 dilution of anti-RyR (clone sc-13942, Santa Cruz).

### Calcium imaging

Intracellular free Ca^2+^ measurements were performed in diaphragmatic myotubes grown on coverslips using the ratiometric probe Fura-2 (5 µM), and frames were collected at 2 s intervals for a period of 100 s. A customized system was employed for KCl perfusion and pharmacologic reagents applied to myotubes as follows: 10 µM CFTR-inh172 (Calbiochem) for 30 min; 2 µM thapsigargin (Sigma) for 400 s; 100 µM ryanodine (Sigma) for 10 min; 50 µM 2-aminoethoxydiphenylborate (APB) (Calbiochem) for 30 min; and 10 µM U73122 (Sigma) for 20 min.

### RT–PCR

Murine CFTR primers contained the following sequences (5′ to 3′): TCTCTGCCTTGTGGGAAATC (forward), and AGTACCCGGCATAATCCAAG (reverse); human CFTR primers consisted of the following (5′ to 3′): CTACATGGAACACATACCTTCG (forward), and GGTGATAATCACTGCATAGC (reverse); GAPDH primer sequences (5′ to 3′) were: AGCAATGCCTCCTGCACCACC (forward), and CCGGAGGGGCCATCCACAGTC (reverse). Real-time PCR of CFTR expression was performed with FAST SYBR Green, and relative quantification was determined using the cycle threshold method (StepOnePlus, Applied Biosystems). Muscle Ring Finger 1 (MuRF-1) and atrogin-1 expression were quantified according to the manufacturer (TaqMan).

### RNase protection assay

^32^P-labelled riboprobes were synthesized using commercial mouse multiprobe kits (BD Biosciences), protected RNA fragments were separated using a 5% polyacrylamide gel, and detected by autoradiography. Bands of individual mRNAs were quantified and normalized to the L32 housekeeping gene (FluorChem 8000, Alpha Innotech).

### ELISA and western blot assays

An immunoassay kit was used to quantify NF-kB (p65 subunit) within nuclear extracts, according to the manufacturer's instructions (Trans-AM, Active Motif). To inhibit the NF-kB pathway, myotubes were treated with the IKK-2 inhibitor SC-514 (Calbiochem) at a dose of 100 µM [52], applied one hour prior to cytomix/LPS stimulation. Western blotting was used to evaluate activation of ERK1/2 MAPK, using antibodies against the total and phosphorylated forms of the protein (Cell Signaling).

CFTR protein expression was evaluated in lysates from human skeletal muscle tissue, E6/E7 human muscle cells, Calu3 cells, and HEK cells with stable overexpression of CFTR. CFTR was immunoprecipitated with anti-CFTR antibody (clone M3A7) and anti-mouse Ig beads. The precipitates were separated using an 8% polyacrylamide gel, and then analyzed by western blotting with a second anti-CFTR antibody (clone 23C5-2) and the TrueBlot detection system (eBioscience).

### *Pseudomonas aeruginosa* lung infection

Mice were infected by intratracheal inoculation at 1×10^5^ cfu using a mucoid strain encapsulated within agar beads, and euthanized at day 2 post-infection, as we have previously described in detail [53].

### Diaphragmatic contractile function

Diaphragms were mounted in an organ bath with oxygenated Krebs solution as previously described in detail [53]. The force-frequency relationship was determined by sequential supramaximal electrical stimulation for 1 s over a range of stimulation frequencies. In separate experiments, diaphragmatic contraction was induced by exposure to a modified Krebs solution containing 60 mM KCl (37°C, pH 7.40).

### Animals and human materials

Animal procedures were done in accordance with the guidelines established by the Canadian Council on Animal Care, and were approved by the McGill University Animal Care Committee. Pulmonary infection studies involved male mice at approximately 10 weeks of age, consisting of CFTR^tm1UNC^ mice and their wild-type littermates (*Cftr^−/−^* and *Cftr^+/+^*, respectively) on the C57Bl/6J background. The *Cftr^−/−^* and *Cftr^+/+^* littermate control mice used for generating primary muscle cell cultures were on the C57Bl/6J or Balb/c background. All mice were generated through breeding of heterozygous *Cftr^+/−^* mice maintained under specific pathogen-free conditions at the McGill University Research Institute animal facilities. Human muscle biopsy tissue was obtained from brain-dead organ donors after approval by the McGill University human ethics committee.

### Statistical analysis

All data are presented as mean values±SEM. Group mean differences were determined by Student's t-test or analysis of variance, with post hoc application of the Tukey test where appropriate. A statistics software package was used for all analyses (SigmaStat 2.0, SPSS). Statistical difference was defined as p<0.05.
